# Solvent-thermal approach of MIL-100(Fe)/Cygnea/Fe_3_O_4_/TiO_2_ nanocomposite for the treatment of lead from oil refinery wastewater (ORW) under UVA light

**DOI:** 10.1038/s41598-024-54897-x

**Published:** 2024-02-23

**Authors:** Wahid Zamani, Saeedeh Rastgar, Aliakbar Hedayati, Mohsen Tajari, Zahra Ghiasvand

**Affiliations:** 1 Department of Environmental Science, Faculty of Natural Resources, University of Kurdistan, Sanandaj, 15175-66177 Iran; 2 Department of Environmental Sciences, Faculty of Fisheries and Environmental Sciences, Gorgan University of Agricultural Sciences and Natural Resources, Gorgān, 49189-43464 Iran; 3 Faculty of Fisheries and Environmental Sciences, Gorgan University of Agricultural Sciences and Natural Resources, Gorgān, 49189-43464 Iran; 4 Department of Fisheries, Bandargaz Branch, Islamic Azad University, Bandargaz, 48731-97179 Iran; 5 Department of Animal Science and Aquaculture, Faculty of Agriculture, Dalhousie University, Halifax, Canada

**Keywords:** Oil refinery wastewater, Organic–metallic framework, Core–shell structure, Photocatalyst, TiO_2_, Biochemistry, Chemical biology, Environmental sciences, Chemistry

## Abstract

The main purpose of this research endeavor is to reduce lead concentrations in the wastewater of an oil refinery through the utilization of a material composed of oyster shell waste (MIL-100(Fe)/*Cygnea*/Fe_3_O_4_/TiO_2_. Initially, iron oxide nanoparticles (Fe_3_O_4_) were synthesized via solvent-thermal synthesis. It was subsequently coated layer by layer with the organic–metallic framework MIL-100 (Fe) using the core–shell method. Additionally, the solvent-thermal method was utilized to integrate TiO_2_ nanoparticles into the magnetic organic–metallic framework’s structure. Varieties of analytical analysis were utilized to investigate the physical and chemical properties of the synthetic final photocatalyst. Nitrogen adsorption and desorption technique (BET), scanning electron microscopy (SEM), scanning electron diffraction pattern (XRD), and transmission electron microscopy (TEM). Following the characterization of the final photocatalyst, the physical and chemical properties of the nanoparticles synthesized in each step, several primary factors that significantly affect the removal efficiency in the advanced oxidation system (AOPs) were examined. These variables consist of pH, photocatalyst dosage, lead concentration, and reaction temperature. The synthetic photocatalyst showed optimal performance in the removal of lead from petroleum wastewater under the following conditions: 35 °C temperature, pH of 3, 0.04 g/l photocatalyst dosage, and 100 mg/l wastewater concentration. Additionally, the photocatalyst maintained a significant level of reusability after undergoing five cycles. The findings of the study revealed that the photocatalyst dosage and pH were the most influential factors in the effectiveness of lead removal. According to optimal conditions, lead removal reached a maximum of 96%. The results of this investigation showed that the synthetic photocatalyst, when exposed to UVA light, exhibited an extraordinary capacity for lead removal.

## Introduction

As a result of Iran’s abundant hydrocarbon reserves, the oil and gas industry possesses distinct importance and priority. Due to inadequate management, the majority of the hazardous substances that contaminate the petroleum wastewater produced during the cleaning of oil containers enter the environment^1^. The presence of heavy metal contamination in sediments has a detrimental impact on indigenous aquatic organisms. The principal apprehension pertaining to these pollutants is the potential for the development of cancer or genetic mutations^2^. Water pollution and the extinction of aquatic and terrestrial species result from the release of hydrocarbon pollutants containing heavy metals^3^. This obstruction prevents the exchange of oxygen between the atmosphere and the aquatic environment, depletes soil fertility, and drastically reduces dissolved oxygen levels^4^. The long-term exposure of living organisms to these compounds can lead to bioaccumulation. The discharge of hydrocarbon waste contaminated with heavy metals into water sources results in depleted soil fertility and increased dissolved oxygen levels in the water. These factors hinder the transfer of oxygen from the atmosphere to the aquatic environment^5^. The removal of heavy metals from petroleum residue has necessitated the use of a variety of techniques, as conventional cleansing methods are insufficient. Despite their effectiveness, each of these approaches has flaws^6^. It is better to use heterogeneous photocatalysis with semiconductors as catalysts in environmental applications because they work more efficiently, are easier to make, and can be extracted from water in different ways^7^. In addition to their capacity to generate charge carriers through the conversion of light energy to chemical energy, these substances exhibit additional attributes that render them well-suited for photocatalytic reactions. Among these are optical and catalytic properties^8^. TiO_2_ and ZnO are, according to the majority, two of the most photoactive catalysts^7^. Their disadvantages, which include a high rate of photogenerated electron–hole pair recombination and a low sensitivity to irradiation due to their broad energy bandgap, limit their applicability^9^. To get around all of this, we need to make new, efficient, multifunctional semiconductors with great photocatalytic properties, the right rates of photogenerated pair recombination, and a narrow bandgap. The primary drawbacks associated with titanium dioxide photocatalysts are their substantial specific area and high light absorption^10^. Increasing the stability of titanium dioxide on a filter consequently improves its ability to obstruct light. As a consequence of the absorber's absorption of organic molecules, this phenomenon develops: an increase in the concentration of organic compounds in the vicinity of the catalyst particles^11^. Using a magnetic photocatalyst also makes it easy to separate the catalyst from the wastewater, which cuts down on waste and makes the process more effective at getting rid of organic pollutants. It is important to acknowledge that the choice of base has an impact on both the optical activity and efficiency of titanium dioxide^12^.

Scholarly publications have documented instances where the optical activity of titanium dioxide was observed to decrease as a result of its thermal interaction with the base. As a consequence of the catalyst's absorptive characteristics, an elevated concentration of organic molecules is generated in the vicinity of the catalyst particles. Additionally, the utilization of a magnetic adsorbent facilitates a more efficient separation of the catalyst from the wastewater^13^. This results in improved organic pollutant removal and decreased refuse production. It is crucial to understand that the base choice affects the optical activity and efficacy of titanium dioxide. Indeed, instances have been documented where the optical activity of titanium dioxide was observed to be diminished as a result of its thermal interaction with the base^14^. The present study signifies the inaugural inquiry into the fabrication and assessment of the performance of a nanocomposite material composed of Fe_3_O_4_/MIL-100(Fe)/TiO_2_. An organic–metallic framework was employed to fabricate a titanium dioxide nanocomposite structure. The objective of the research was to employ the characteristics of the nanocomposite to efficiently eliminate lead contaminants from petroleum wastewater for the purpose of environmental remediation. The photocatalyst that was synthesized exhibits the capacity to efficiently remove heavy metal-containing compounds that are frequently encountered in diverse industrial waste streams. Photocatalytic removal systems have the potential to operate as viable substitutes for traditional purification systems.

## Materials and methods

### Materials

The chemicals used in this study were of high purity and obtained from reputable suppliers. Iron (III) chloride hexahydrate extra pure 98.0% assay (FeCl_3_⋅6H_2_O, Sinochem, China), terephthalic acid for synthesis (H_2_BDC), tetra-n-butyl orthotitanate for synthesis (TBOT, Merck, Germany), sodium silicate solution extra pure (Merck, Germany), n-hexane hypergrade 99% (Merck, Germany), and hydrochloric acid (HCl, Merck, Germany), as well as ethanol and methanol absolute 99.9% (C_2_H_5_OH, Scharlou, Spain), were used without further purification. Both the synthesis and processing procedures include the use of deionized water. Lamp (UVA, 6W, Philips) was used.

### Collection shell of (Andonata Cygnea)

The photocatalyst synthesis involved the utilization of oyster shells as a precursor material. These oyster shells were sourced from a dumpsite located in Tajan River State, Mazandaran, Iran, without any cost incurred. The shells underwent a meticulous washing process with hot water in order to eliminate any contaminants^15^. Subsequently, the material underwent calcination at a temperature of 900 °C for a duration of 3 h within a muffle furnace. Next part, the subsequent procedure involved subjecting the specimen to a drying process within an oven set at a temperature of 180 °C. The desiccated powder underwent many washes using methanol at a concentration of 99%, as well as alcohol and distilled water. The ultimate photocatalyst was acquired subsequent to undergoing a drying process under an acidic environment, characterized by a pH value of 5^16^.

### Synthesis of titanium dioxide nanoparticles

A solution was prepared by dissolving 10 ml of tetrabutyl orthotitanate (TBOT) in 60 ml of 2-propanol. The resulting mixture was then subjected to agitation at room temperature using a magnetic stirrer for 10 min. A total volume of three milliliters of deionized water was incrementally added, dropwise^15^. The milky solution was subjected to stirring using a magnetic stirrer for a duration of two h. After undergoing a 5 min separation process using centrifugation at a speed of 4500 revolutions per minute, the resultant mixture was subjected to numerous washes using 2-propanol. The sediment was later treated to calcination for a length of 4 h at a temperature of 500 °C, after complete desiccation^17^.

### Synthesis of MIL-100(Fe)

0.5 gr of iron powder were mixed with 50 ml of deionized water and an acidic mixture made up of 626 µl of hydrofluoric acid (HF) at a concentration of 48% and 566 µl of nitric acid (HNO_3_) at a concentration of 65%. This produced the MIL-100 alloy nanoparticles. The solution was subjected to stirring for a duration of 15 min at room temperature (25 °C), utilizing a magnetic stirrer^18^. After the introduction of 1.37 g of crystalline powder comprising 1, 3,5-benzene tricarboxylic acid (H_3_BTC), the mixture underwent magnetic stirring for an additional duration of 30 min, or until achieving complete homogeneity. The resultant mixture was placed in a 50-ml Teflon stainless steel autoclave and subjected to heating at a temperature of 150 °C for a duration of 24 h^19^. After the autoclave had undergone the cooling process, the final solid product, characterized by its vibrant orange hue, was extracted with a centrifuge and afterwards subjected to rinsing with deionized water. In order to remove any remaining unreacted compounds, the sediment obtained was exposed to heating operations lasting 3 h at temperatures of 80 °C in water and 60 °C in ethanol^20^.

### Synthesis of Fe_3_O_4_

The mixture obtained was placed in a Teflon-coated stainless steel autoclave with a volume of 50 ml. The autoclave was then subjected to a temperature of 150 °C for a duration of 24 h in an oven^9^. After the autoclave had undergone the cooling process, the resultant solid product, characterized by its vibrant orange hue, was extracted by means of a centrifuge and afterwards subjected to rinsing with deionized water. The resultant mixture was placed in a 50-ml Teflon stainless steel autoclave and subjected to heating at a temperature of 150 °C for a duration of 24 h in an oven^21^. After the autoclave had undergone the cooling process, the final solid product, characterized by its vibrant orange hue, was extracted by means of a centrifuge and afterwards washed with deionized water. In order to remove any remaining unreacted compounds, the final sediment was exposed to thermal treatments lasting 3 h at temperatures of 80 °C in water and 60 °C in ethanol^22^.

### Functionalization of Fe_3_O_4_

Fe_3_O_4_ particles were modified using the next method, which involved using the organic molecule methacrylic acid (MAA). By following the steps outlined above, 0.05 g of Fe_3_O_4_ that had been made was mixed with a 10 ml solution of methacrylic acid dissolved in ethanol that contained 0.29 mmol^23^. The compound was subjected to continuous agitation at ambient temperature for a duration of 24 h. After the separation of the functionalized Fe_3_O_4_ particles using an external magnetic field, they were subjected to numerous rinses with ethanol and distilled water. Subsequently, the sample underwent dehydration at a temperature of 70 °C^24^.

### Synthesis of nanocomposite Fe_3_O_4_/MIL-100(Fe)

Synthesis magnetic nanoparticles Fe_3_O_4_/MIL-100 by adding Fe_3_O_4_ nanoparticles one layer at a time. A total duration of 15 min was allocated to the process of dissolving 0.3 g of functionalized Fe_3_O_4_ nanoparticles in a solution of iron chloride, which was then dissolved in 25 ml of ethanol with a concentration of 10 mol^25^. Subsequently, the sample was distributed for a duration of 30 min in a solution containing 25 ml of 1,3,5-benzene tricarboxylic acid (H_3_BTC) dissolved in 10 mmoles of ethanol. To prevent the formation of nanoparticles, both operations were carried out at a temperature of 70 °C, therefore mitigating their buildup. During each transitional phase, the nanoparticles were subjected to purification and isolation utilizing ethanol and a magnetic field. The sample underwent desiccation in a vacuum oven at a temperature of 70 °C and a pressure of 10 mbar^26^.

### Synthesis of nanocomposite Cygnea/Fe_3_O_4_/MIL-100(Fe)/TiO_2_

**Figure 1:**
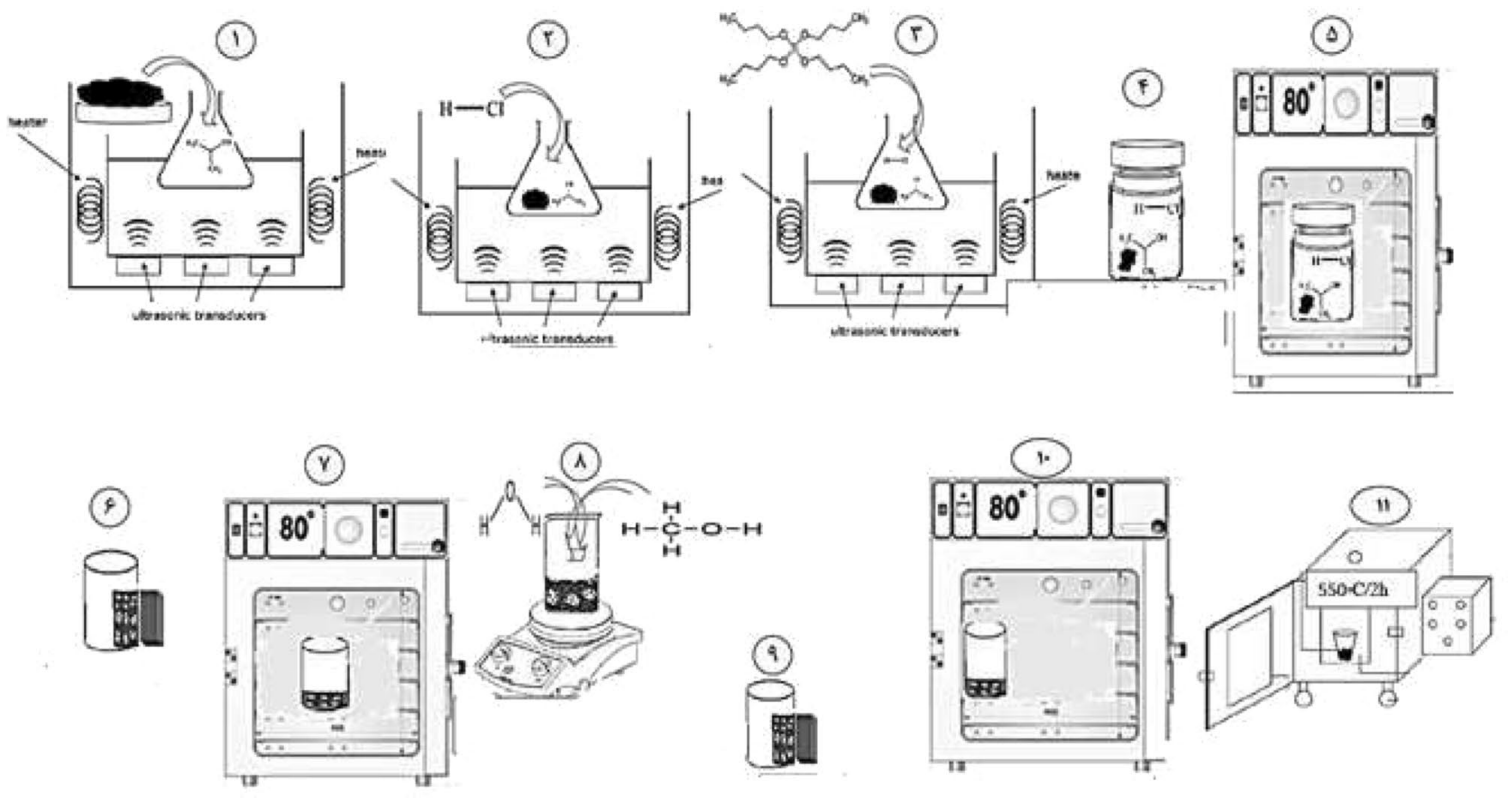
Synthesis of nanocomposite* Cygnea/*Fe_3_O_4_/MIL-100(Fe)/TiO_2_.

To completely separate the particles, 25 mg of the Fe_3_O/MIL-100 nanoparticles that were made in the previous step were mixed with 50 ml of ethanol using an ultrasonic chamber for 15 min (1 and 2) (Fig. 1). After adding 250 µl of tetrabutyl orthotitanate (TBOT), the solution was exposed to ultrasonic waves for a duration of 10 min (3 and 4)^27^. Following the addition of 105 µl of HF acid and 3.5 ml of deionized water, the solution waas subjected to ultrasonic vibrations at 25 °C for an additional 20 min (5 and 6). The suspension was subsequently transferred to a 50-ml autoclave made of Teflon-coated stainless steel. The substance was subjected to a temperature of 180 °C for a duration of 12 h (7 and 8)^15^. After the autoclave had cooled down to the surrounding temperature, the resulting product was retrieved using a magnet and underwent several ethanol rinses to remove any remaining unreacted compounds. The final powder was obtained by subjecting it to a vacuum of 10 ml and dehydrating it for a duration of 12 h at a temperature of 70 °C (9, 10 and 11)^15^.

### Characterization of the Fe3O4/MIL-100(Fe)/TiO2

Field emission scanning electron microscope (FESEM, TESCAN, MIRA ll, Czech Republic), high-resolution transmission electron microscope (HR-TEM, FEI, TEC9G20, 200kv, America), X-ray diffraction analysis (XRD, Rigaku-Ultime IV, Japan), and Fourier transform infrared spectrometer (FTIR, Thermo Nicolet Avatar 370, USA) were applied to characterize the size and the functional groups on the surface of the photocatalyst^25^. Raman spectroscopy (UniDRON–UniNanoTech, South Korea), Energy dispersive X-ray spectroscopy (EDX, TESCAN, MIRA II, France) were employed for the analysis of the photocatalyst surface. According to the Scherer equation, the crystallite size was computed using the following equation^28^:

1 $$ {\text{D}} = \frac{k \lambda }{{\beta \cos \theta }} $$

where *D* is the crystallite particle size (nm), *k* (= 0.89) is the Scherer constant, *λ* denotes the X-ray wavelength, *β* is the full width of the peak at half-maximum intensity (FWHM), and *θ* shows the diffraction angle^16^.

The elemental composition and oxidation states of elements and the frequency distribution of the elements were specified using MAP analysis (TESCAN, MIRA II, France). The thermal characteristics of the photocatalyst under heating environments were investigated using the thermo-gravimetric analysis/differential thermo-gravimetric (TGA-DTG, SDT-Q600-V20.9 Build 20-USA)^15^. The surface area analysis, thermodynamic size, and surface charge of *Fe*_*3*_*O*_*4*_*/MIL-100(Fe)/*TiO_2_ were determined by Brunauer–Emmett–Teller (BET, BELSORP MINI II, BEL PREP VAC II, Japan). Diffuse reflectance spectroscopy (DRS, S 4100, SCINCO, South Korea) to measure the characteristic reflectance spectrum, and the point of zero charges (pzc) was utilized^29^.

### Characteristics of FFW

The FFW was collected from fish farms in Golestan, Gorgan, Iran, and stored at 4 °C (Table 3). Chemical oxygen demand (COD), biochemical oxygen demand (BOD), nitrite and nitrate, PO_4_^3–^-P, DO, electrical conductivity (EC), turbidity, total suspended solids (TSS), volatile suspended solids (VSS), total dissolved solids (TDS), total Kjeldahl nitrogen (TKN), SO_4_^2−^, and chloride were analyzed utilizing APHA standard procedures^9^ .

### Photocatalytic removal experiments

The suspension was subsequently transferred to a 50-ml autoclave made of Teflon-coated stainless steel. The substance was subjected to a temperature of 180 °C for a duration of 12 h. After the autoclave had cooled down to the surrounding temperature, the resulting product was retrieved by employing a magnet and underwent several ethanol rinses to remove any remaining unreacted compounds^30^. The final powder was obtained by subjecting it to a vacuum of 10 ml and dehydrating it for a duration of 12 h at a temperature of 70 °C. The specimen was exposed to UV radiation under ambient conditions, and the percentage of complete elimination was calculated. Additional parameters underwent similar optimization techniques. The efficacy of removal by Fe_3_O_4_ nanoparticles and Fe_3_O_4_/MIL-100 (Fe) nanostructure was also evaluated under UV light after determining the optimal circumstances^31^. The primary objective of this study is to investigate the characteristics of a 6 w Philips UVA lamp, which has specific dimensions of 302.5 mm in length and 16 mm in diameter, along with a current intensity of 425 milliamperes. The photoreactor was effectively mixed using an air compressor, which serves as the main mechanism for generating active radicals OH^15^. The time intervals at which samples were obtained were as follows: 0, 180, 120, 60, 40, 20, and 240 min. In all of the experiments, the reaction mixture was left in the dark for 20 min without any UV light on. This was done to reduce the chance that the photocatalyst would absorb the substance instead of removing it. This time frame was officially classified as the dark period^32^.

## Results

### Characterization

#### FESEM

**Figure 2:**
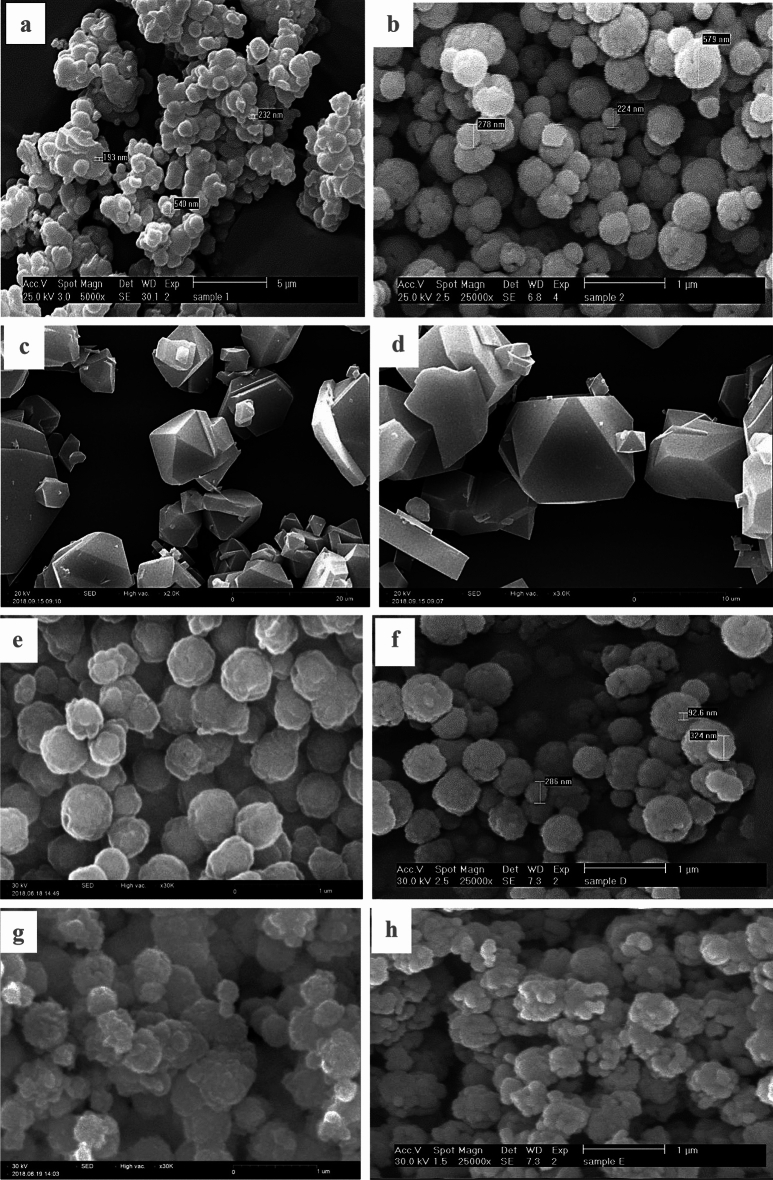
SEM analysis of (**a**) TiO_2_ nanoparticles(*5 µ , (**b**) Fe_3_O_4_ (*1 µm), (**c**) and (**d**) organic–metallic framework MIL-100(Fe) (*20 µm), (**e**) and (**f**) nanocomposite Fe_3_O_4_/MIL-100 (Fe) and (**g**) and (**h**) Fe_3_O_4_/MIL-100(Fe)/TiO_2_ (*1 µm).

Figure 2 showed various magnifications of the synthesis process. The figure includes scanning electron microscope (SEM) images obtained from nanoparticles of titanium dioxide and iron oxide, as well as the MIL-100 (Fe) organic–metallic framework. Additionally, it features the core–shell structure of Fe_3_O_4_/MIL-100(Fe) and the photocatalyst Fe_3_O_4_/MIL-100(Fe)/TiO_2_. The first image in Fig. 2a depicts the titanium dioxide nanoparticles that were synthesized in order to ensure their integration into the final composition of the photocatalyst^15^. Figure 2b indicate various magnifications of the synthesis process. The figure includes scanning electron microscopy (SEM) images obtained from nanoparticles of titanium dioxide and iron oxide, as well as the MIL-100 (Fe) organic–metallic framework^33^. Additionally, it features the core–shell structure of Fe_3_O_4_/MIL-100(Fe) and the photocatalyst Fe_3_O_4_/MIL-100(Fe)/TiO_2_. The first image in Fig. 2c depicts the titanium dioxide nanoparticles that were synthesized in order to ensure their integration into the final composition of the photocatalyst. Figures 2d and e correspond to the organic–metallic framework MIL-100 (Fe), exhibiting an octahedral shape and characterized by its crystalline nature and absence of impurities, as seen by the photographs^34^. The visual representation showcases particles of diverse diameters, including those that are on the nanoscale scale. The nanocomposite, Fe_3_O_4_/MIL-100 (Fe), which consists of a magnetized organic–metallic framework, is visually shown in Fig. 2f–h^35^.

The provided photos demonstrate that the organic–metallic framework saw notable changes in surface roughness and particle size after interacting with the iron oxide nanoparticles while still retaining its spherical form^36^. The SEM Fig. 2g and h of the Fe_3_O_4_/MIL-100(Fe)/TiO_2_ nanocomposite show that the titanium dioxide nanoparticles are spread out evenly and form clusters on the surface of the magnetized organic–metallic framework structure after TiO_2_ is added^35^. The substance has undergone a significant transformation from its original spherical shape to a nearly spherical mass. When comparing the particle size and shape of Fe_3_O_4_/MIL-100 (Fe)/TiO_2_ nanocomposite to Fe_3_O_4_/MIL-100 (Fe), there are clear differences. This is evident from the reduced detectability of nanoparticles with sizes below 300 nm in photos (e) and (f)^37^.

#### TEM analysis

**Figure 3:**
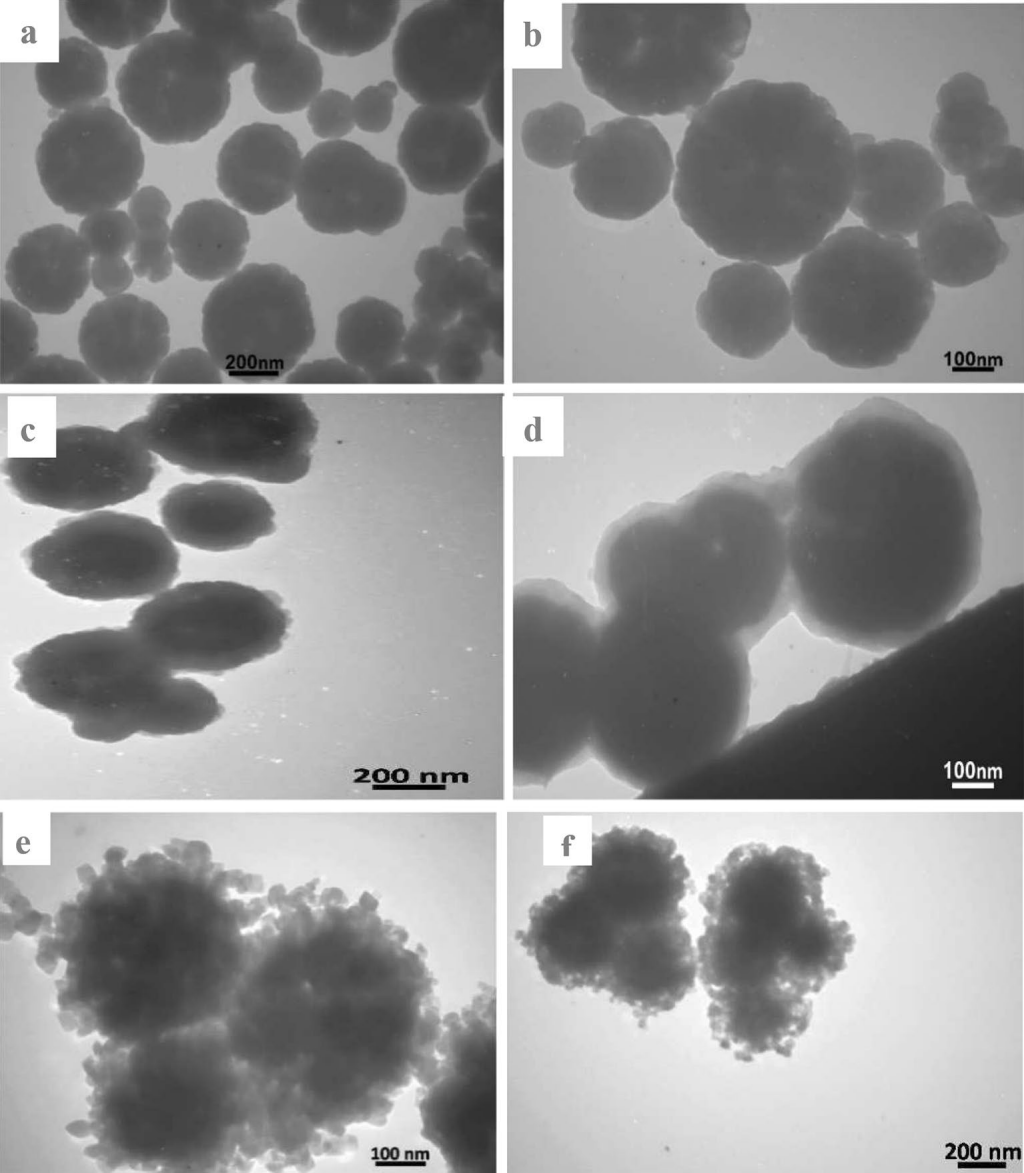
TEM analysis of (**a** and **b**) Fe_3_O_4_, (**c** and **d**) Fe_3_O_4_/MIL-100(Fe) nanocomposite and (**e** and **f**) Fe_3_O_4_/MIL-100(Fe)/TiO_2_ photocatalyst in two different magnifications.

The standard error of the mean (SEM) for the Fe_3_O_4_/MIL-100(Fe)/TiO_2_ nanocomposite indicates that the distribution of titanium dioxide (TiO_2_) nanoparticles is uniform, with the formation of clusters observed on the surface of the magnetized organic–metallic framework structure subsequent to the addition of TiO_2_ (Fig. 3a and b)^34^. It is indisputable that the material has experienced a substantial alteration from its initial spherical configuration, culminating in the development of a mass that is nearly spherical. Compared to Fe_3_O_4_/MIL-100 (Fe), the Fe_3_O_4_/MIL-100 (Fe)/TiO_2_ nanocomposite has particles that are much smaller and have different shapes (Fig. 3c and d)^36^. The diminished detectability of nanoparticles with dimensions below 300 nm may be shown in (Fig. 3e and f), as supported by the data Iron oxide nanoparticles can be observed. The particles possess a nanoscale organic–metallic lattice around them. The utilization of ultrasonic bath methodology results in the formation of an organic–metallic framework that encapsulates iron oxide nanoparticles, exhibiting consistent thickness and shape^15^. This feature indicates the confirmation of the core–shell structure of the Fe_3_O_4_/MIL-100 (Fe) nanocomposite. The ultrasonic bath method is used to make an organic–metallic framework that surrounds iron oxide nanoparticles and has a consistent thickness and shape. The core–shell structure of the Fe_3_O_4_/MIL-100 (Fe) nanocomposite is confirmed by this characteristic^17^. This coating layer containing TiO_2_ nanoparticles by solvent-thermal method has a uniform shape and size, and as it is clear in the pictures, the dimensions of the photocatalyst have increased slightly after the deposition of titanium dioxide nanoparticles^24^.

#### XRD analysis

**Figure 4:**
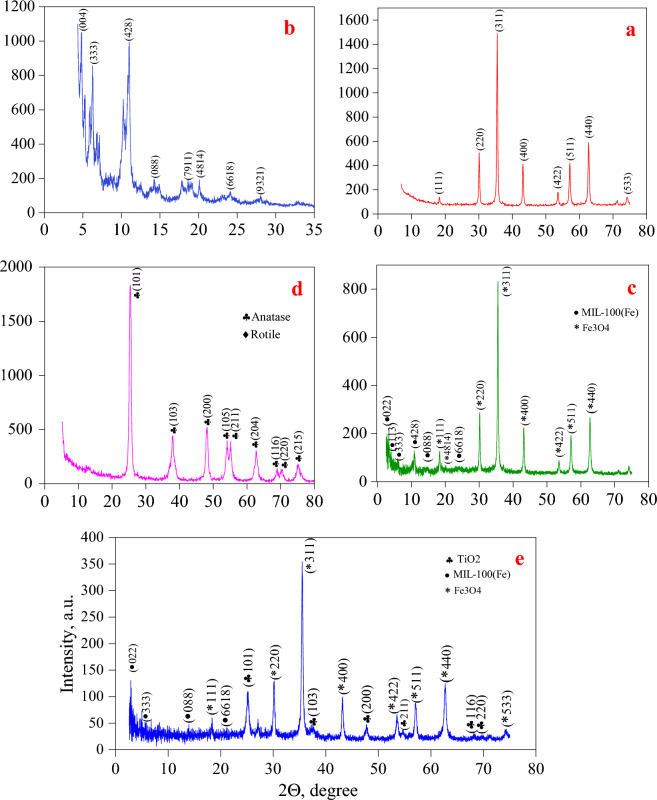
XRD analysis of (**a** and **b**) Fe_3_O_4_, (**c** and **d**) Fe_3_O_4_/MIL-100(Fe) nanocomposite and (**e** and **f**) Fe_3_O_4_/MIL-100(Fe)/TiO_2_ photocatalyst in two different magnifications.

X-ray diffraction analysis was employed to examine the morphology and crystal structure of Fe_3_O_4_ nanoparticles, the organic–metallic framework MIL-100 (Fe), and the synthetic photocatalyst Fe_3_O_4_/MIL-100(Fe)/TiO_2_. As depicted in Fig. 4a illustrates the diffraction pattern of Fe_3_O_4_ nanoparticles. The peaks observed in this pattern can be attributed to the cubic crystal structure featuring centered faces of Fe_3_O_4_ nanoparticles, as reported by the JCPDS^38^. It was seen that the diffraction pattern of Fe_3_O_4_ nanoparticles has clear peaks at 2ϴ values of 18.34°, 30.1°, 35.5°, 43.1°, 53.5°, 57°, 62.7°, and 66°. The pattern to the signs in the corresponding database, namely (511), (440), (111), (220), (311), (400), (422), and (533), respectively^39^. The Fig. 4b illustrates the diffraction pattern of MIL-100 (Fe) organic–metallic framework nanoparticles, as determined by X-ray diffraction analysis. The diffraction patterns of nanoparticles synthesized in the current study reveal the following values in 2ϴ: (333) 6.24°, (428) 11°, (088) 14.2°, (7911) 18.6°, (4814) 20°, (6618) 24.07° and (9321) 27.9°^40^. The findings of this study indicate that the organic–metallic framework was synthesized and designed successfully. Following the successful synthesis of MIL-100 (Fe) nanoparticles, this organic–metallic framework was contemplated as the foundation for producing the ultimate Fe_3_O_4_/MIL-100 (Fe)/TiO_2_ photocatalyst and stabilizing TiO_2_ nanoparticles^41^. To achieve this, Fe_3_O_4_ nanoparticles were layered with these nanoparticles to magnetize the organic–metallic framework and facilitate the capture of the final photocatalyst from the aqueous environment (Fig. 4c)^42^. Figure 4d illustrates the XRD pattern of Fe_3_O_4_/MIL-100 (Fe) nanoparticles. According to the results of Dong et al.^41^ and Kerli et al.^42^ the appearance of the main peaks characteristic of the crystal structure of the organic framework MIL-100(Fe) metal. In addition to the peaks of Fe_3_O_4_ nanoparticles, confirms the successful synthesis and matrix stability of these nanoparticles after 21 cycles of placing them on Fe_3_O_4_ nanoparticles in the Fe_3_O_4_/MIL-100(Fe) structure synthesized in this research^41,42^. The X-ray diffraction pattern of TiO_2_ nanoparticles is depicted in Fig. 4d, where the principal peak, which is indicative of the anatase phase of TiO_2_, is situated at 25.37°^15^. Additionally, the XRD pattern delineated in this investigation reveals it. The peaks appearing at 2θ are about (101) 25.37°, (103) 36.95°, (200) 48.15°, (105) 54.1°, (211) 55.15° and (204) 65.62°, indicates the formation of anatase phase of TiO_2_ nanoparticles^43^. In accordance with the results of Zamani et al.^15^, and according to the above, the presence of the TiO_2_ anatase phase index peak at 2θ of about 25.37°. In the diffraction pattern of Fe_3_O_4_/MIL-100 nanocomposite (Fe)/TiO_2_ in Fig. 4e in the present research, confirms the formation of anatase phase of TiO_2_ nanoparticles in the structure of photocatalyst Fe_3_O_4_/MIL-100(Fe)/TiO_2_. Crystal size of anatase phase of titanium dioxide and iron dioxide observed in the respective XRD spectra was estimated by Scherer’s equation at 2θ peaks of 25.37° and 35.5°, respectively. Based on this equation, the average crystal size for the anatase phase of titanium dioxide, iron dioxide, Fe_3_O_4_/MIL-100(Fe) nanoparticles, and the final photocatalyst is 15.29°, 31.96°, 31.87°, and 29.51°, respectively (Fig. 4e)^36^.

#### TGA and DTG analysis

**Figure 5:**
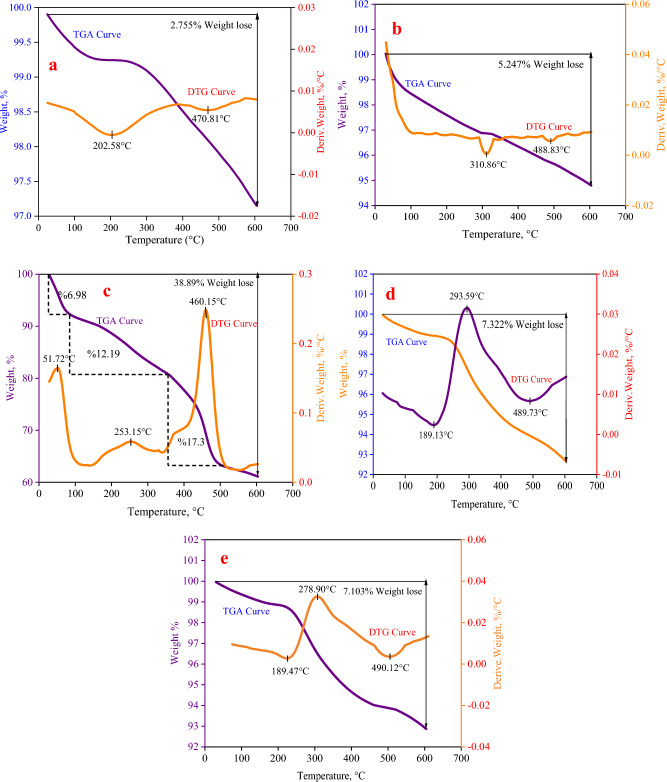
TGA-DTG analysis related to (**a**) Fe_3_O_4_, (**b**) TiO_2_, (**c**) organic–metallic framework MIL-100(Fe).

A thermogravimetric analysis (TGA) was done on Fe_3_O_4_, TiO_2_, MIL-100 (Fe), and Fe_3_O_4_/MIL-100 (Fe)/TiO_2_ nanocomposites to find out how stable they are at high temperatures and how they are arranged on the surface of Fe_3_O_4_ particles (Fig. 5a and b). The study was performed under controlled conditions within an argon environment, with temperatures varying between 25 and 600 °C^44^. The findings obtained are illustrated (Fig. 5c and d). To summarize, the first decline in mass observed between temperatures of about 25 and below 100 °C, specifically at a temperature of 51 °C, is commonly ascribed to the liberation of unbound water molecules from both the structural framework and the pores^15^. The second drop occurred within a temperature range of around 100–350 °C, with a specific temperature of 253 °C. The decrease in intensity seen can be ascribed to the condensation of water or OH molecules, which are coordinative bound to the core Fe(III) atom. The ultimate decrease, which accounts for a larger percentage of the total decrease, occurred at a temperature of 460 °C^45^. The weight of the sample is determined by the researcher and serves as an indicator of the degree of decomposition of organic compounds inside the crystal structure, as well as the possibility of a full breakdown of H_3_BTC^46^. The verification of the existence of an organic–metallic framework on the surface of iron oxide particles may be established by the observation of a weight reduction of 7.32% in the Fe_3_O_4_/MIL-100(Fe) nanocomposite, as illustrated (Fig. 5e). This weight loss is consistent with the weight reduction of 2.75% indicated in the figure pertaining to iron oxide (a)^45^. The graph depicts the relationship between weight loss and the moisture content of the sample while exposed to a temperature of 189 °C. Following this, the reduction in weight caused by the condensation of OH molecules inside the structure of the sample is detected at a temperature of 293 °C^47^. The third weight loss occurs at a temperature of 489 °C, and this decline may be ascribed to the degradation of organic compounds inside the crystal lattice of the organic-metal framework. Diagram (e) depicts three separate stages of weight reduction, which align with the initial moisture content (189 °C), condensation of water molecules (OH) (278 °C), and disintegration of the organic–metallic framework (490 °C), as seen (Fig. 5d)^48^. Based on the result that pure TiO_2_ didn’t reduce weight much, it is thought that the weight loss percentage of the photocatalyst Fe_3_O_4_/MIL-100 (Fe)/TiO_2_, which is made by adding TiO_2_ nanoparticles to the core–shell structure of Fe_3_O_4_/MIL-100 (Fe)/TiO_2_, would be lower than that of the magnetized organic-metal framework shown in the picture below^49^.

#### BET analysis

**Figure 6:**
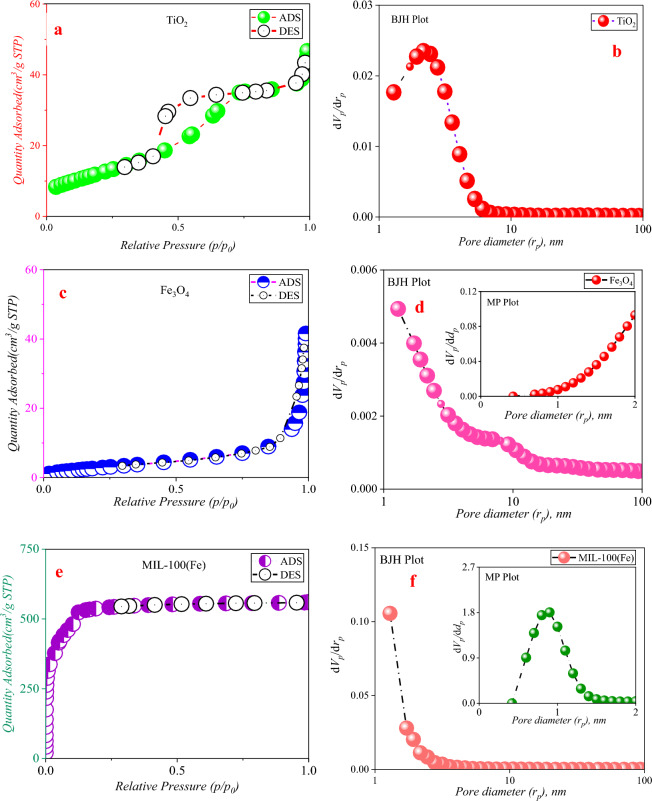

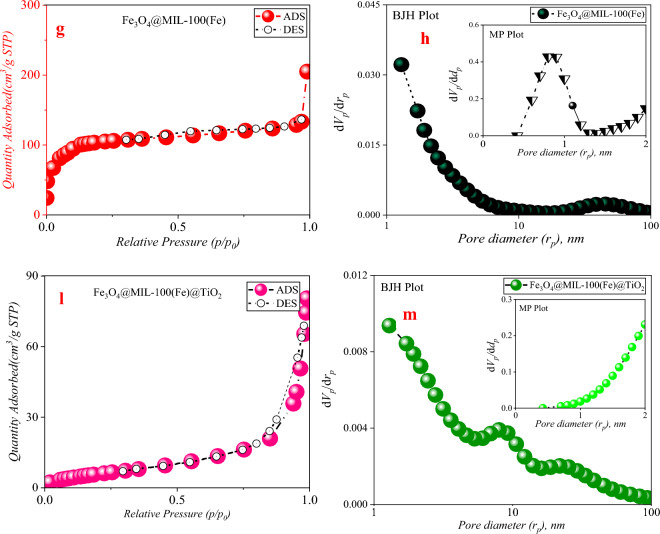
Nitrogen absorption/desorption curves, BJH and MP-Plot of samples synthesized at 77 K.

Figure 6a depict the nitrogen adsorption and desorption curves, as well as the particle size distribution. The IUPAC classification categorizes physical absorption isotherms into six separate types based on their corresponding cavity configurations (Fig. 6b)^50^. Based on the IUPAC classification, the absorption/desorption curve of nitrogen gas for TiO_2_ has features consistent with isothermal type IV behavior, with some indications of type V behavior, implying that the specimen primarily consists of interstitial spaces (Fig. 6c)^51^. Furthermore, a small fraction of micro-holes can be observed, and the resulting residual ring provides evidence of the existence of this phenomena (Fig. 6d). Figure 6e and f depict the nitrogen adsorption and desorption curves as well as the particle size distribution. The IUPAC classification categorizes physical absorption isotherms into six separate types based on their corresponding cavity configurations^15^. Based on the IUPAC classification, the absorption/desorption curve of nitrogen gas for TiO_2_ has features consistent with isothermal type IV behavior, with some indications of type V behavior, indicating that the specimen primarily consists of interstitial spaces. Furthermore, a small fraction of micro-holes can be observed, and the resulting residual ring provides evidence of this phenomena (Fig. 6g)^52^. Based on the MP-Plot diagram, it has been shown that the particle size distribution inside this framework is less than 2 nm (Fig. 6h)^53^. The synthetic organic–metallic framework was determined to possess pores characterized by a specific surface area of 1955 cm^2^/g and an average diameter of 0.8 nm, as determined using BET analysis^54^. The organic–metallic framework synthesized in this investigation exhibited a total pore volume of 0.993 cm^3^/g, as reported by Sedaghat et al.^55^ and Kiyani et al.^56^. Notably, this value surpassed the findings reported by the aforementioned researchers^55,56^.

Based on the findings, the aggregate void volume inside the synthesized organic–metallic framework was determined to be 0.993 cm^3^/g. This measurement aligns with the outcomes reported by Wu et al.^57^ and Ji et al.^58^, surpassing their respective values. The nitrogen absorption and desorption diagram associated with iron oxide likewise conforms to the type III isotherm in terms of its shape, often suggesting a lack of porosity in these particles (Fig. 6I)^59^. The iron oxide core–shell composite, covered with an organic–metallic framework, exhibits a nitrogen absorption/desorption isotherm that adheres to the type I diagram^60^. This observation suggests that the microspore structure plays a crucial role in determining the characteristics of this composite material. Conversely, the MP-Plot diagram illustrates that the structure exhibits a higher degree of complexity^61^. Additionally, it provides an analysis of the particle size distribution within the microporosity range. Furthermore, the core–shell combination exhibited a distinct specific surface area of 380.79 cm^2^/g (Fig. 6m)^62^. It can be asserted that a significant transformation of the pores has occurred, whereby the majority have transitioned into micropores. This alteration may be attributed to the substantial deposition of an organic–metallic framework possessing a microspore structure onto the surface of iron oxide^63^. The nitrogen adsorption/desorption diagram of the Fe_3_O_4_/MIL-100(Fe)/TiO_2_ nanocomposite displays some resemblance to the type V isotherm, while the presence of a residual loop in the figure indicates otherwise^64^. The accompanying BJH chart essentially shows that it exhibits a discernible level of mesoporosity. The narrow residual ring that was detected is consistent with the H_3_ classification, suggesting the existence of slit-like pores inside the sample^65^. The deposition of TiO_2_ nanoparticles on the Fe_3_O_4_/MIL-100 (Fe) nanocomposite has resulted in a reduction in the specific surface area. This drop may be attributed to the obstruction of certain pores and channels within the organic framework. Metal is positioned on the surface of iron oxide^65^. This observation indicates that the produced photocatalyst is not a mere aggregation of TiO_2_ and Fe_3_O_4_/MIL-100 (Fe) nanoparticles. Instead, it demonstrates that the TiO_2_ particles are uniformly distributed over the surface and cavity structure of the Fe_3_O_4_/MIL-100 (Fe) nanocomposite (Fig. 6a–m)^63^. Table 1 provides a summary of the structural data associated with the samples, namely the specific surface area (S_BET_), total volume (V_total_), and average diameter of holes (dp) (Table 1).

**Table 1:** Structural characteristics of the synthesized samples.

Sample	Structural parameters						
	S_BET_, m^2^/g	V_mic_, cm^3^/g	V_mes_, cm^3^/g	V_total_, cm^3^/g	R(d_p_) nm	V_mic_, %	V_mes_, %
TiO_2_	44.31	0.051	0.076	0.127	2.45	40.15	59.84
MIL-100(Fe)	1955	0.899	0.094	0.993	0.8	81.06	18.9
Fe_3_O_4_	11.28	0	0.064	0.064	1.29	–	–
Fe_3_O_4_/MIL-100(Fe)	380.79	0.176	0.099	0.176	2	–	–
Fe_3_O_4_/MIL-100(Fe)/TiO_2_	23.81	0	0.129	0.129	1.29	–	–

#### VSM analysis

**Figure 7:**
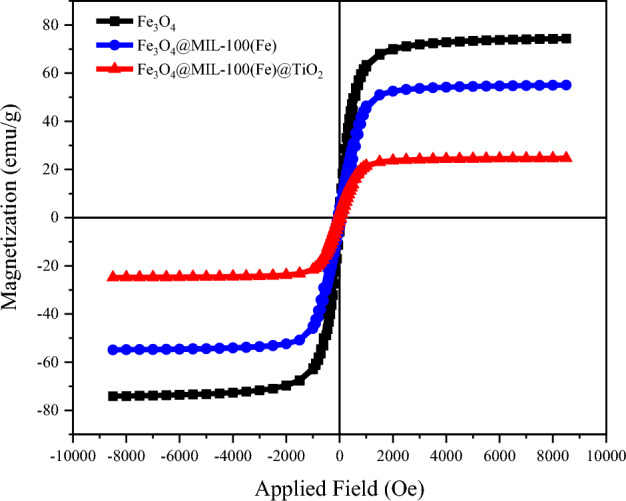
Vibrational magnetometric analysis of three magnetic samples Fe_3_O_4_, Fe_3_O_4_/MIL-100(Fe) and Fe_3_O_4_/MIL-100(Fe)/TiO_2_ in an applied field of 1 Tesla.

This observation indicates that the synthesized photocatalyst is not a mere amalgamation of TiO_2_ and Fe_3_O_4_/MIL-100 (Fe) nanoparticles. Furthermore, it can be observed that the TiO_2_ particles are uniformly distributed on both the surface and cavity structure of the Fe_3_O_4_/MIL-100 (Fe) nanocomposite (Fig. 7)^66^. Table 1 provides a summary of the structural data associated with the samples, namely the specific surface area (S_BET_), total volume (V_total_), and average diameter of holes (dp). When compared to iron oxide, the organic–metallic magnetic framework Fe_3_O_4_/MIL-100(Fe) has less saturation magnetization after 24 h^67^. This decline can be attributed to the inclusion of the diamagnetic material MIL-100(Fe). The observed decrease in composition from Fe_3_O_4_/MIL-100(Fe) to Fe_3_O_4_/MIL-100(Fe)/TiO_2_ can also be attributed to the inclusion of the diamagnetic titanium dioxide. The absence of the residual loop in the sample indicates that it possesses superparamagnetic^15^.

#### FTIR analysis

**Figure 8:**
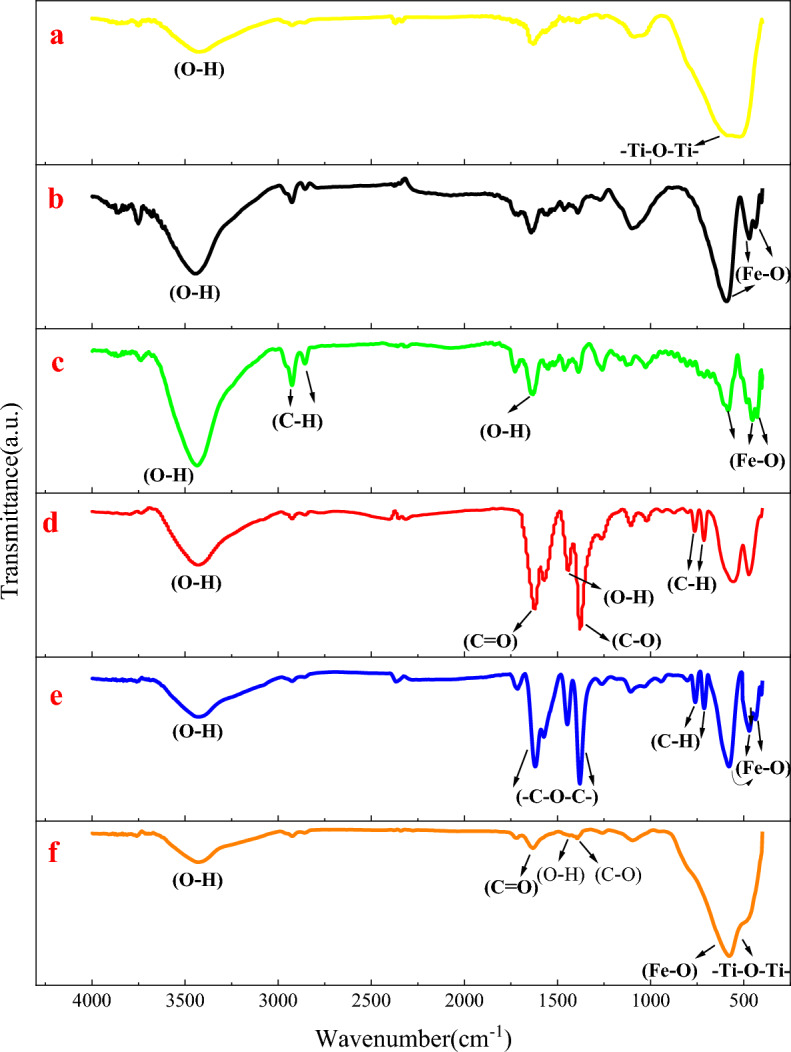
FTIR spectrum of the synthesized photocatalyst.

To further characterize the structure of the synthesized nanoparticles, FT-IR spectroscopy was utilized. As depicted in Fig. 8a–f. The peaks observed at 1443/cm and 1622/cm in diagram (d) correspond to C=O stretching vibrations and O–H wave vibrations, respectively (Fig. 8a)^68^. The peak at 1377/cm is also associated with C–O stretching vibrations, most likely as a result of the shifts in vibrational energy from approximately 1100–1337/cm caused by the bonding of C–O with Fe (C–O–Fe) (Fig. 8b)^69^. Consequently, the organic component of the organo-metallic framework is associated with all three peaks that signify the existence of the carboxyl group. Specifically, the two peaks observed at 713/cm and 762/cm can be ascribed to the C–H stretching vibrations of the benzene ring within this framework (Fig. 8c). These findings align entirely with those of Rastgar et al.^29^ corresponds. Carboxylic O–H may also account for the broad peak at 1556/cm. In accordance with the findings of Zhao et al.^70^ and Torres et al.^71^, the absence of a peak in the range of 1710–11,720/cm suggests C=O stretching vibrations associated with H_3_BTC. Moreover, this suggests that no residual H_3_BTC was detected in the final sample wash because of the synthesis method being executed appropriately. Diagram (e) illustrates that the main peaks signifying Fe_3_O_4_, such as 436/cm, 1470/cm, and 593/cm, are associated with the Fe–O stretching vibrations in Fe_3_O_4_ (Fig. 8d)^71^. The presence of all the mentioned peaks corresponds to the symmetric and asymmetric stretching and bending vibrations of the COO-framework-metal group. It verifies the successful synthesis of the core–shell structure Fe_3_O_4_/MIL-100(Fe)^70^. The peak observed at approximately 3434/cm in the samples is attributable to the hydroxyl (OH) group and O–H stretching vibrations of water molecules adsorbed on the sample’s surface^16^. Figure 8f depicts the sole distinct peak of the 576/cm band, which corresponds to the –Ti–O–Ti structure and, as shown in diagram (a), the –Ti–O–Ti bond within the TiO_2_ nanoparticles’ structure (Fig. 8e). The broadening of the 593/cm peak of Fe–O due to its overlap with this peak indicates the presence of the TiO_2_ phase in the photocatalyst and the formation of a bond within this structure^72^. Conversely, fainter peaks associated with the carboxyl group (–O–C–O–) are detected. Based on supplementary examinations of this specimen, including SEM, TEM, and others, this concern may suggest that the nanoparticles have been successfully incorporated into the Fe_3_O_4_/MIL-100(Fe) structure, resulting in the formation of the Fe_3_O_4_/MIL-100(Fe) structure of TiO_2_^70^. In diagram (c), the subject is MAF-Fe_3_O_4_. It is observed that the intensity of the 593/cm peak, which corresponds to the Fe–O bond, decreases after Fe_3_O_4_ functionalization. The peaks detected at 2926/cm and 2856/cm are indicative of the stretching vibrations associated with the C–H band of the CH_2_ group, which is linked to methacrylic acid (Fig. 8f)^73^. Furthermore, the broad peak observed at approximately 3434/cm can be attributed to the overlapping O–H stretching vibrations of water molecules that were absorbed onto the surface of the sample. O and are constituents of the molecular structure of methacrylic acid^72^.

#### pH effect

**Figure 9:**
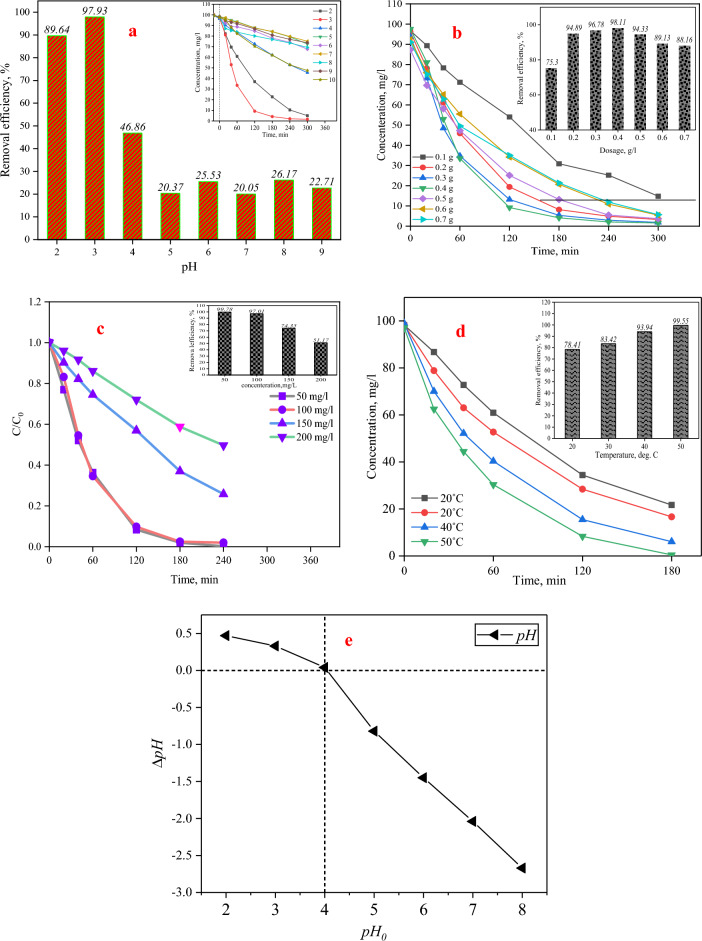
Effect of (**a**) pH, (**b**) photocatalyst dosage, (**c**) time, (**d**) Temprature, (**e**) zero charg point.

The pH of the solution plays a crucial role in the photocatalytic degradation of wastewater since it significantly influences the rate of wastewater removal. A lot of things can be changed by the pH of the reaction solution, including the size of the catalyst particles, where the conduction bands are located, and how the wastewater attaches to the surface of the semiconductor. The pH parameter exerts a significant influence on both the photocatalytic degradation mechanism and the degradation rate of the wastewater^74^. Positive holes are the primary oxidation species in the lower pH range, whereas hydroxyl radicals (OH^−^) prevail in the upper or neutral pH range, governing the oxidation process^15^. An alkaline solution possesses the capacity to generate radicals. However, the presence of electrostatic repulsion might impede the absorption of anions by surface sites on semiconductors, resulting in a decrease in the efficiency of wastewater degradation. Radical generation can occur in an alkaline solution^75^. Conversely, the efficacy of wastewater degradation may be hindered by electrostatic repulsion in cases where semiconductor surface sites are unable to adsorb anions from the wastewater. The participation of hydroxyl radicals plays a vital role in the process of photocatalytic degradation of N-azoid wastewaters^55^. This degradation process occurs through an interconnected network facilitated by the presence of TiO_2_. According to the results obtained from this study, the degree of ORW removal at a particular time point was most pronounced under conditions of low pH, especially at a pH of 3, and decreased as the pH level climbed^29^. The observed behavior can be attributed to a catalytic property that exhibits extraordinary sensitivity to the presence of H^+^ ions. The catalyst often acquires a negative surface charge when OH^−^ ions are absorbed, particularly under conditions of alkaline pH^76^. The experiment demonstrated that the best effectiveness of removal was attained at an acidic pH. This can be attributed to the superior performance of OH radicals under acidic conditions. Moreover, it can be shown that an increase in pH leads to a corresponding enhancement in the rate of the polymerization reaction^9^. The presence of a variety of organic compounds can be observed in ORW wastewater as a result of many parameters, including composition type and surface load, among others. Several chemicals possess the capacity to exist in positive, neutral, and negative states inside aqueous solutions. The presence of variables at different pH levels may exert varying effects on the process of photocatalytic degradation of these compounds^32^. In this study, a total duration of 240 min was allocated for the purpose of UV light irradiation. The pH levels encompassed a range spanning from 2 to 9. The experimental conditions included a photocatalyst dosage of 0.02 mg/l and a temperature of 25 °C^15^. The percentage of wastewater clearance is significantly influenced by the pH, as seen in Fig. 9a. A non-linear rise in the proportion of wastewater discharge was noted with a fall in pH levels from 9 to 3.

Numerous studies have employed point-zero charge (PZC) titanium dioxide (TiO_2_) to investigate the impact of pH on the performance of photocatalytic oxidation. The absence of electrostatic force at the point of zero TiO_2_ charge results in minimal interaction between the photocatalyst particles and the organic pollutant^77^. However, at a pH value below the point of zero charge (PZC), the photocatalyst’s absorption surface acquires a positive charge, enabling it to function as both an ion absorber and a cation repeller. Conversely, when the pH exceeds zero, the adsorption surface acquires a negative charge, transforming it into a cation absorber while simultaneously repelling anions. Some organic compounds that are negatively charged can stick to TiO_2_ in a polar way^16^. This could be the basis for later photocatalytic phases. Mousavi et al.^9^ showed that changes in pH can be explained by the point of zero charge and how it relates to the photocatalyst's surface charge. The graph of the zeta potential and zero charge point of the photocatalyst synthesized in this investigation is depicted in Fig. 9a. The precise value of this point for the synthetic photocatalyst was 4 ± 0.04, which signifies the equilibrium between the positive and negative electric charges present on its surface^15^. TiO_2_ demonstrates amphoteric characteristics at a pH range of 4 ± 0.04. In acidic environments with a pH value higher than pH, electron–hole formation enhances the absorption of anions. As per the following Eqs. (2) and (3), the surface of the catalyst will be positively charged at a pH below 4 ± 0.04 or in an acidic solution; conversely, the opposite holds true^78^:

2 $$ {\text{TiOH}}_{{({\text{Surface}})}} + {\text{H}}^{{ + }{}} \to {\text{TiOH}}_{{{2}\;\;\;({\text{surface)}}}}^{ + } \;\;\;({\text{pH}} < {\text{ PZC}}) $$

3 $$ {\text{TiOH}}_{{({\text{Surface}})}} + {\text{OH}}^{ - } \to {\text{TiO}}^{ - }_{{\left( {{\text{Surface}}} \right)}} + {\text{H}}_{{2}} {\text{O}}\;\;\; ({\text{pH}} > {\text{ PZC}}) $$

Most of the removal happens at low pH levels because red reactive wastewater 195 has a negative charge and is negatively charged. It has an electrostatic tendency to stick to the surface of TiO_2_ when it comes into contact with acidic environments^73^. However, the high concentration of H^+^ at pH values below 2 can impede the rate of the reaction. The observed issue may be attributed to the aggregation of TiO_2_ particles under low or acidic pH conditions, which is a property that can also diminish the catalyst’s specific surface area, which is crucial for photon absorption and wastewater absorption^9^ (Fig. 9e).

#### Effect of photocatalyst dose

Figure 9d depict the relationship between the dose of photocatalyst and the percentage of ORW elimination. The range of photocatalyst concentrations employed in this study varied from 0.01 to 0.07 g/l, and the highest duration of UVA light irradiation was 240 min. The temperature of the system was recorded as 25 °C, and further adjustments were made to regulate the pH level to 3. As seen in Fig. 9b, the removal efficiency demonstrated a gradual improvement from 85.35 to 98.11% with the augmentation of the photocatalyst dose from 0.01 to 0.04 g/l. A reduction in magnitude was found subsequent to an escalation in dosage from 0.04 to 0.07 g/l. To mitigate the occurrence of catalyst wastage and excessive use, it is imperative to ascertain the optimal dose for effective removal of ORW^9^. The photocatalytic degradation rate exhibits an upward trend with an increase in the quantity of photocatalyst. However, above a certain threshold, additional increments in the amount of photocatalyst lead to a decline in the degradation process^15^. There are three elements that may contribute to this observed tendency. The introduction of an extra amount of photocatalyst to a system where all wastewater molecules have already been integrated onto its surface does not provide any additional enhancement in the performance of the photocatalytic process^16^.

An additional factor that may contribute to this phenomenon is the excessive presence of photocatalyst particles within the reaction solution, resulting in increased turbidity in the suspension. Consequently, this elevated turbidity hinders the pace of degradation^79^. Also, as the concentration of the photocatalyst went up, there was a big increase in the number of particle collisions and contacts inside the solution. The issue at hand could make active molecules less effective because they interact with titanium dioxide particles in their natural state, which would make wastewater degradation less effective^80^. The tendency for aggregation and clustering exhibited an upward trend with the escalation of the dose of the photocatalyst. The rate of photocatalytic degradation was seen to decrease when the accessible surface area for light absorption was reduced^79^. Consequently, the passage of light and the extent of photocatalyst exposure to UV radiation were diminished. Increasing the dosage of the photocatalyst resulted in the fragmentation of the active sites. This phenomenon effectively inhibited the generation of more electron–hole pairs^17^. However, when the quantity of photocatalyst employed is below the optimal level, it restricts both the surface area of the photocatalyst and its capacity to absorb light. Moreover, the decrease in degradation percentage seen at greater concentrations may be ascribed to the deactivation of the active molecules upon contact with the ground-state molecules^77^. Based on the aforementioned interpretations, it was concluded that the most effective dosage was 0.04 g/l, leading to a clearance rate of 98.11% during a time span of 240 min. The obtained results are consistent with previous reputable studies. Numerous studies have provided evidence of a negative link between the degradation efficiency and the dose of photocatalyst, if it is above a certain threshold^63^.

#### Effect of initial wastewater concentration

This studies was conducted using four concentrations, specifically 150, 100, 50, and 200 mg/l. The findings of the study revealed that the greatest degree of ORW removal, amounting to 97.01%, was achieved when the concentration of the substance was set at 100 mg/l and the pH level was maintained at 3. Additionally, a dosage of 0.04 mg/l was employed to achieve these results. The influence of the initial wastewater concentration on the rate of removal is seen in Fig. 9c. Previous studies have demonstrated the correlation between the augmentation of wastewater concentration and the rate constant (k) of decolonization. In tests done at four different concentrations (150, 100, 50, and 200 mg/l), the results showed that when the conditions are perfect (pH = 3 and dosage = 0.04 mg/l), getting rid of ORW at a concentration of 50 mg/l for 240 min was amazingly effective at 99.78% (Table 2)^9^. Likewise, when the concentration reached 100 mg/l after a duration of 240 min, the degree of ORW removal was found to be 97.01%. Therefore, the percentage of elimination has exhibited no significant alteration despite the twofold increase in concentration^33^. Subsequently, a decrease in the degradation rate was seen with an increase in the concentration of the ORW. Before reaching a certain threshold, the effectiveness of degradation showed an increasing tendency as the concentration of ORW increased^19^. An elevated initial ORW concentration may enhance the likelihood of a reaction occurring between ORW molecules and oxidizing species. However, it concurrently diminishes the efficacy of ORW degradation^65^.

**Table 2:** Characteristics of ORW.

Characteristics		Untreated value	Treated value	Efficiency (%)
pH^a^	ORW	7.47	6.74	–
Total COD (TCOD)^b^	ORW	46,125.65	821.22	98.21
Total BOD (TBOD)^b^	ORW	16,543.13	498.43	97.82
Total dissolved solids (TDS)^b^	ORW	9761.87	723.11	92.59
Total suspended solids (TSS)^b^	ORW	5643.21	422.12	92.51
Volatile suspended solids (VSS)^b^	ORW	4965.22	1121.65	77.40
Total kjeldahl nitrogen (TKN)^b^	ORW	3765.33	913.44	75.74
SO_4_^2−b^	ORW	2187.54	543.29	75.16
PO_4_^3−^-P^b^	ORW	1.9987	0.9821	50.86
Cl^−b^	ORW	14.76	7.23	51.01

^a^,  dimensionless; ^b^,  R195/l.

This happens because more wastewater stops the production of OH radicals on the catalyst's surface because ORW ions take up active sites. Another possible explanation for these results could be the impact of UV intensity on the ORW itself. In situations where the ORW concentration is high, the ORW molecules, rather than the TiO_2_ particles, may absorb a significant portion of ultraviolet radiation^23^. Consequently, the effectiveness of the catalytic reaction diminishes as the concentration of reactive species, specifically O_2_ and the OH radical, declines^29^. Numerous studies have provided support for the aforementioned findings. The primary deterioration takes place inside the response zone, which is located in close proximity to the irradiated area and experiences a significantly greater level of radiation intensity compared to other places^24^. The presence of higher ORW concentrations leads to a decrease in the amount of light that can penetrate, resulting in a reduction in degradation at longer distances from the light source or the reaction zone. It may be deduced that there exists a direct relationship between the initial concentration of the ORW and the quantity of catalyst needed for degradation (Table 2)^25^.

#### Temperature

**Figure 10:**
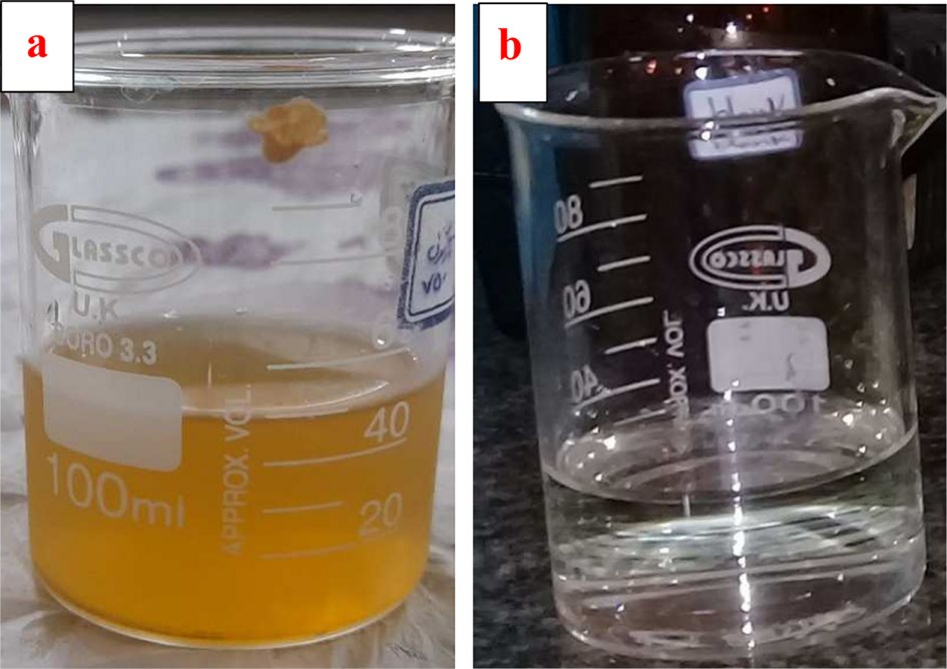
ORW treatment , (**a**) befor, (**b**) after use of photocatalyst.

Temperature is a significant and pivotal aspect in the examination of the photocatalytic degradation of ORWs. The research findings indicate that semiconductor photocatalysis is not solely dependent on temperature (Fig. 10a). However, raising the temperature generally promotes and strengthens the reaction by hindering the recombination of electron–hole pairs, hence speeding the process^23^. After optimizing the dosage of the photocatalyst and adjusting the reaction pH, further tests were carried out at temperatures of 20, 30, 40, and 50 °C in order to investigate the influence of temperature and thermodynamic factors on the process. The tests were done autonomously, according to the specified conditions: a pH level of 3, a photocatalyst dosage of 0.04 g/l, and a duration of 240 min for UV radiation exposure. The correlation between temperature and the extent of ORW loss is seen in Fig. 9d The findings suggest that there was an increase in the quantity of removal by the Fe_3_O_4_/MIL-100 (Fe)/TiO_2_ photocatalyst as the temperature increased throughout the stipulated time period^77^. Consequently, there was a reduction in the maximum time allotted for removal, from 240 to 180 min. Therefore, it may be argued that the degradation process exhibits endothermic characteristics, given that the reaction rate increases with an elevation in temperature^37^. Figure 10b displays the removal of ORW by a spectrophotometer across the wavelength range of 400–700 nm, under optimal conditions. These conditions include a pH of 3, a concentration of 100 mg/l, a photocatalyst dose of 0.04 g/l, a temperature of 35 °C, and a duration of 240 min of UV light exposure.

#### Potential Fe_3_O_4_/MIL-100(Fe)/TiO_2_ recycle

**Figure 11:**
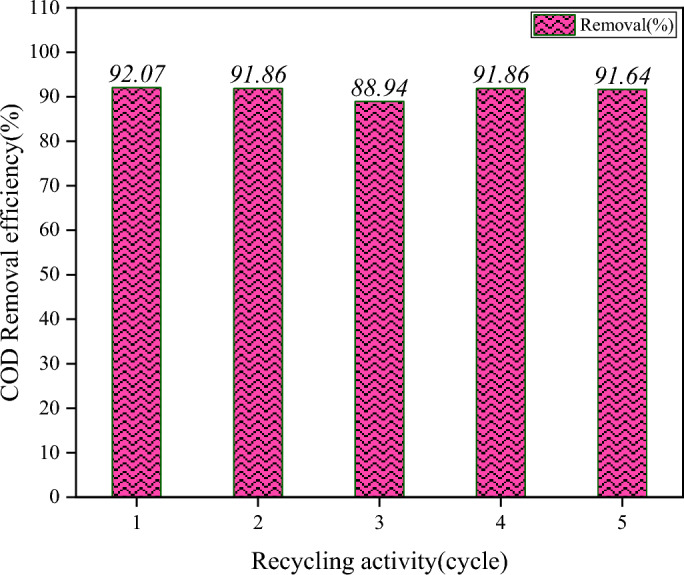
COD removal as a function of the number of wastewater treatment cycles by photocatalyst Fe_3_O_4_/MIL-100(Fe)/TiO_2_ (pH 3, concentration 100 mg/l, photocatalyst dose 0.04 g/l, temperature 35 °C and UV light exposure time equal to 240 min).

To get the best removal rate, a full study was done on the main factors that have a big effect on how well the advanced oxidation system (AOPs) works at getting rid of things. The variables considered in this study encompassed reaction temperature, pH level, dose of photocatalyst, ORW concentration, as well as the physical and chemical characteristics of the nanoparticles produced at each stage and the resulting photocatalyst^16^. Based on the data collected, it was seen that the Fe_3_O_4_/MIL-100(Fe)/TiO_2_ photocatalyst was the best at getting rid of lead and cadmium under certain conditions. These settings included a pH of 3, a wastewater content of 100 mg/l, a dose of 0.04 g/l, and a temperature of 35 °C. Moreover, the photocatalyst exhibited a significant level of reusability even after enduring five successive cycles^10^. The presence of these chemicals hindered the efficient absorption of organic pollutants, resulting in a decrease in the efficiency of the photocatalyst. After undergoing the third cycle of purification, the photocatalyst was extracted and subsequently exposed to a four-hour calcination procedure at a temperature of 550 °C^56^. This treatment was performed in preparation for the reuse of the photocatalyst in the fourth cycle. Following the completion of the fourth round, the photocatalyst was extracted and subsequently reapplied during the fifth cycle. The process for reusing the photocatalyst was similar throughout the original and subsequent versions^9^. As seen in Fig. 11 (cycles four and five), the process of calcination effectively reinstated the photocatalyst to its original level of activity. Hence, to rejuvenate and reinstate the photocatalytic functionality and eliminate any organic substances that could have infiltrated the catalyst's pores, the process of calcination becomes necessary^79^. According to the findings of this inquiry, it has been determined that the synthetic photocatalyst exhibits stability and retains its initial activity during numerous cycles of purification.

#### Coexisting ions

The presence of coexisting ions in wastewater typically impedes the adsorption of heavy metal ions that are deleterious in nature. The presence of common negatively charged species, such as sulfate, nitrate, phosphate, and carbonate, could have a big effect on the adsorption of pb^81^. Conversely, coexisting cations may exhibit a synergistic adsorption effect when it comes to the removal of lead. A lot of research has been done on this topic, but the molecular mechanism that explains how coexisting ions affect platinum (Pb) adsorption by nanofibers is still not clear^82^.

#### Comparison with other photocatalysts

The literature is limited regarding the application of MIL-100 (Fe), *Cygnea*, Fe_3_O_4_, and TiO_2_ in the treatment of wastewater. Comparing the current findings to those that other researchers have recorded (Table 3) reveals a noteworthy trend. The information presented in the table provides confirmation that the present photocatalyst, MIL-100(Fe)/*Cygnea*/Fe_3_O_4_/TiO_2_, is innovative, cost-efficient, readily available, and comparatively effective. The first-time use of Cygnea as a precursor to generate a magnetic photocatalyst and remove phosphate and nitrate compounds from OPW was documented in this study. Moreover, in this investigation, refuse was utilized as a beneficial photocatalyst to diminish the levels of pollutants in farm effluent. This matter is of the utmost importance when viewed through an ecological lens.

**Table 3:** Photocatalytic degration efficiency of other photocatalyst.

Photocatalyst	Pollutante	Maximum degration efficiency (%)	References
Fe_3_O_4_@MoS_2_@mesoporous TiO_2_	Methylene blue (MB), Rhodamine B (RhB), and Tetracycline (TC)	99.4%, 96.5%, and 89.3%	^83^
Sulfur and nitrogen co-doped carbon dots (NSCDs)	Pb(II)	95.8%	^84^
Zero-valent iron/phosphoric/TiO_2_	Pb(II) > Cu(II) > Cd(II)	93%	^85^
(Polyvinyl chloride) PVC/PbO-graphite	Lead (Pb^+2^)	89%	^86^
MWCNTs and TiO_2_	Methyl orange organic	92%	^87^
Hydrogel microcapsules with photocatalytic nanoparticles	Methylene blue (MB)	91%	^88^

### Conclusion

In this study, the solvent-thermal method was used to successfully add TiO_2_ nanoparticles to the magnetic organic–metallic framework’s structure. A variety of analytical methodologies were utilized for evaluating the physical and chemical characteristics of synthetic samples at various phases. It was possible to find MIL-100 (Fe) nanoparticles and TiO_2_ in the anatase phase by looking at the structure of the Fe_3_O_4_/MIL-100 (Fe)/TiO_2_ photocatalyst with XRD. The structure of the photocatalyst also shows carboxyl groups connected to the organometallic framework of MIL-100. A characteristic functional group identification verified that the titanium dioxide nanoparticles (Ti–O–Ti) comprising the structure of the photocatalyst were composed of the proper elements. According to SEM analysis, the photocatalyst’s particles underwent size and shape changes. TEM analysis showed that an organic–metallic framework was being built and that the core of the iron oxide particles had completely broken down. VSM analysis revealed that the samples’ superparamagnetic behavior was caused by the magnetic strength decreasing slowly during the photocatalyst synthesis process. BET analysis was employed to ascertain the specific surface area, pore volume, and isotherm. The study examined the following variables: reaction temperature, pH level, photocatalyst dose, and ORW concentration. Furthermore, the final photocatalyst and the physical and chemical properties of the nanoparticles synthesized at each phase were taken into account. In conclusion, the photocatalyst exhibited its highest level of efficacy in lead removal when subjected to the designated conditions: a pH of 3, a 100 mg/l wastewater concentration, a 0.04 g/l dose, and a temperature of 35 °C. The photocatalyst demonstrated a considerable degree of reusability, even after five consecutive cycles. As a result, the investigations confirmed that the synthesized photocatalyst possessed a significant capacity for eliminating heavy metals from petroleum wastewater. This photocatalyst may be utilized in subsequent research to eliminate additional contaminants from industrial wastewater.

## Data Availability

The data and materials from the current study are available from the corresponding author on reasonable request.
